# Uncommon Complication of a Biliary Leak After Adjustable Gastric Band Removal: A Case Report

**DOI:** 10.7759/cureus.46856

**Published:** 2023-10-11

**Authors:** Elías Ortiz Gómez, Jorge Vera Macías, Manuel A Meza Jasso, José Aldo Guzmán Barba, Isaac Esparza Estrada

**Affiliations:** 1 Bariatric Surgery, Elias Ortiz & Company, Tijuana, MEX

**Keywords:** obesity-related illnesses, spontaneous bile leak, biliary leak, adjustable gastric band complications, bariatric surgery complications

## Abstract

In the context of adjustable gastric band (AGB) placements and the prevalent issue of weight regain with associated complications, revision surgery for gastric bands becomes imperative. Such revisions may encompass band removal or conversion to bariatric procedures, often accompanied by an escalated risk profile, potentially contributing to a 20% morbidity rate. Laparoscopic sleeve gastrectomy (LSG) has gained prominence due to its technical simplicity, effectiveness in weight loss, and lower complication rates. Specific cases involving LSG post-AGB complications are associated with staple line disruptions and leaks.

This case report describes a rare complication in a 59-year-old patient following AGB removal and subsequent laparoscopic sleeve gastrectomy. The complication emerged six hours after the surgery, with approximately 400 cc of bile material reported in the drainage. A laparoscopic reintervention was conducted, revealing bile leakage from the second Couinaud hepatic segment. Successful management of the leakage was achieved through simple hepatic suturing using non-absorbable monofilament. Within 24 hours, no further leakage occurred, and the patient was discharged without additional complications.

Our case also demonstrates how complex it can be to switch between different medical procedures, and it emphasizes the need for careful planning and precise surgery in the evolving world of bariatric medicine. It is worth noting that there is a dearth of literature addressing this specific complication. Consequently, this study has the potential to provide valuable insights for surgeons who may encounter a similar scenario in their clinical practice.

## Introduction

Given the historical context of adjustable gastric band (AGB) placements and the notable incidence of weight regain along with associated complications, revision surgery for gastric bands assumes an essential role in managing such patients. Revision strategies encompass both complete band removal and conversion to alternative bariatric procedures, whether in a single-stage or two-stage approach. It is important to emphasize that these conversions carry an escalated risk profile, including issues like leaks, stenosis, and more, consequently contributing to an increased morbidity rate that can reach up to 20% [1].

Laparoscopic sleeve gastrectomy (LSG) has rapidly gained prominence as a pivotal surgical approach in addressing morbid obesity. As a standalone bariatric procedure, LSG has achieved substantial popularity, emerging as the predominant choice for individuals seeking effective weight management solutions. This ascent has been driven by several factors, including its relative technical simplicity, favorable weight loss outcomes, and a comparatively reduced incidence of complications when compared to other surgical interventions [2,3].

In specific clinical scenarios, LSG is considered an option for managing complications stemming from previous gastric banding procedures. Transitioning from gastric banding to LSG has been associated with a higher probability of disruptions in the staple line, potentially leading to leaks. Notably, the unique complication discussed in this case report aligns with this context, shedding light on the technical challenges associated with transitioning between different weight loss surgeries. This underscores the significance of thorough preoperative planning, precise surgical techniques, and vigilant postoperative monitoring [4].

During our comprehensive literature search, we found no studies referencing the complication highlighted in our report: a biliary leak originating from the second Couinaud hepatic segment. As a result, this likely represents the first documented case detailing this specific complication. To ensure the thoroughness of our investigation, we employed a multifaceted approach, utilizing well-known databases like PubMed, SciELO, UpToDate, and Scopus, as well as a meticulous examination of specialized international scientific journals in the fields of general surgery and bariatrics.

## Case presentation

A 59-year-old patient with a body weight of 136 kg and a BMI of 49 kg/m², diagnosed with systemic arterial hypertension and dyslipidemia, both managed with medical therapy, and a surgical history of adjustable gastric band placement 14 years ago (in 2009), presented to our hospital due to weight regain over the years (exact weight regain is unspecified), seeking adjustable gastric band removal and subsequent LSG.

**Figure 1:**
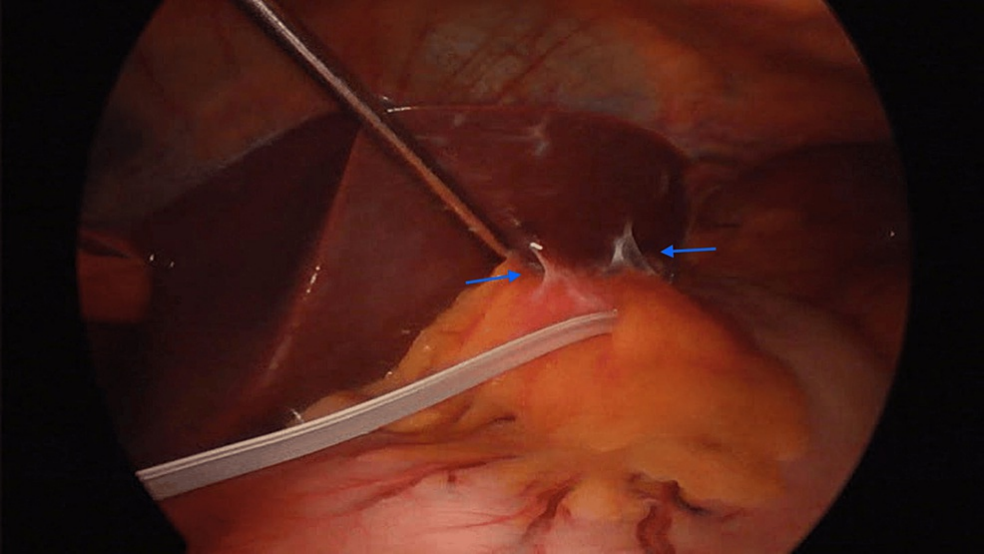
Gastrohepatic adhesions observed prior to gastric band removal

**Figure 2:**
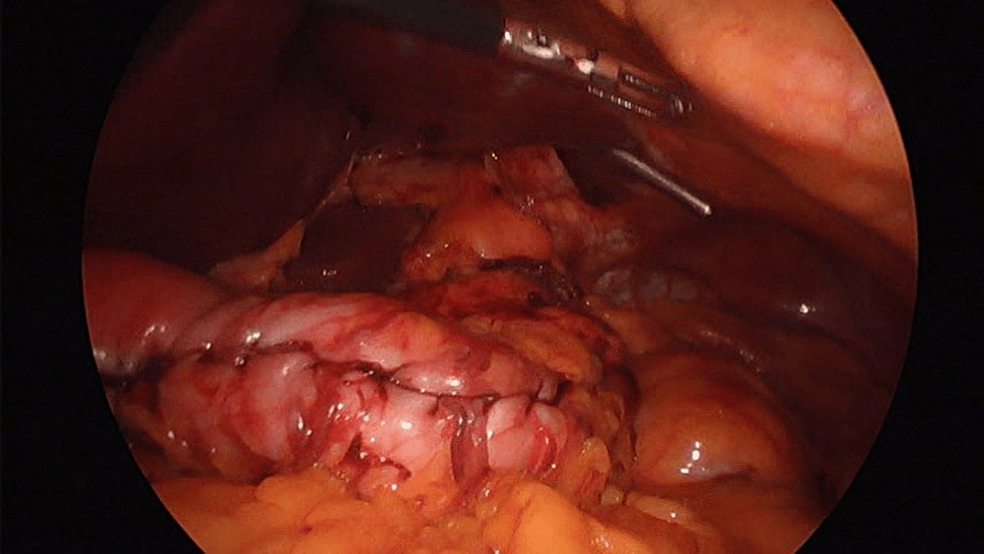
Gastric sleeve performed with stapling line reinforcement

Preoperatively, a diagnostic endoscopy was conducted, revealing no evidence of band erosion. Intraoperatively, gastro-hepatic fibrosis was observed (Figure 1). The gastric band was released by dissecting it away from the liver and stomach, and the fibrous ring was also dissected due to the risk of gastric stenosis. Neither hematic nor biliary fluid leakage was observed, and coagulation of the pre-existing fibrosis was performed. Subsequently, a conventional LSG was performed using a manual stapler, seven black staples, and a 36 Fr calibration tube. The stapling line was further sutured as reinforcement using 2.0 polypropylene sutures (Figure 2). Air and methylene blue leak tests were conducted to ensure the gastric sleeve integrity. A Jackson-Pratt drain was placed as a standard practice, and a postoperative endoscopy revealed no evidence of postoperative bleeding.

**Figure 3:**
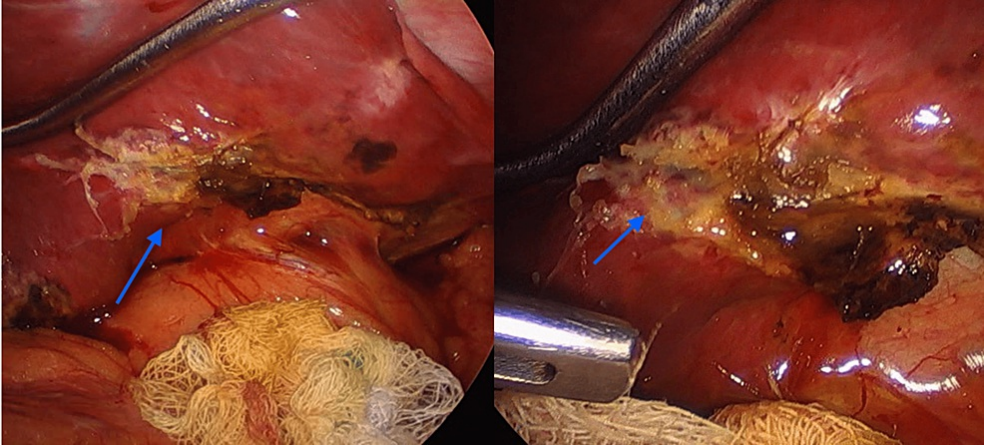
Biliary leak evidenced in the left hepatic lobe (second Couinaud segment)

**Figure 4:**
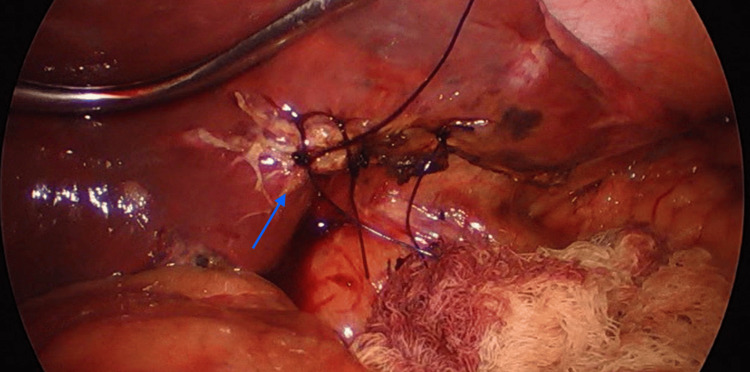
Resolution of the biliary leak with a monofilament suture

Six hours following the surgery, approximately 400 cc of bile material was reported in the drainage. Vital signs remained within normal ranges, but the patient described moderate abdominal tenderness upon palpation. It was determined that a laparoscopic reintervention should be conducted, revealing bile leakage from the second Couinaud hepatic segment (Figure 3). A simple hepatic suturing was performed using non-absorbable monofilament to successfully manage the biliary leakage (Figure 4). Over the subsequent hours, no further biliary material leakage was observed, abdominal pain subsided, and after four days, the patient was discharged without any additional complications. One month post-surgery, the patient experienced a weight loss of 12 kg, remained free of complications, and exhibited proper tolerance to their dietary regimen.

During the in-hospital stay, the patient received medical management for nausea and pain with dexamethasone, metoclopramide, and ketorolac tromethamine, respectively, in addition to an antibiotic regimen of cefazolin.

## Discussion

In line with data released by the American Society for Metabolic and Bariatric Surgery (ASMBS), the utilization of AGB in the United States has undergone a significant decline [5]. This phenomenon can be attributed to the concerning weight regain observed among patients post-AGB placement, coupled with an increased prevalence of enduring postoperative complications. Consequently, this shift in trends has catalyzed a global surge in revisional surgeries, escalating from a mere 10% to a substantial 50% [1].

However, it is imperative to grasp that this paradigm of conversion surgery inherently carries an augmented morbidity and mortality risk profile. This can primarily be attributed to progressive fibrosis within the tissues surrounding the original AGB implantation site, fostering adhesions to various neighboring structures, including the liver, esophageal hiatus, and spleen. These intricate anatomical connections pose a formidable surgical challenge [5].

The complexity associated with converting AGB to LSG in a single procedure is underscored by the multifaceted nature of complications. For instance, Lundeberg et al. documented a notable 20% complication rate in their study [6]. In contrast, a systematic review conducted by Elnahas et al. indicated a relatively broader spectrum of complication rates, ranging from 3% to 15%, for AGB-to-LSG conversions [7]. A parallel study by Falk et al. underscored a 13% short-term complication rate in patients undergoing sleeve gastrectomy after AGB, with issues such as wound infections and postoperative leakage manifesting prominently [8]. Janik et al. enrolled a substantial cohort of 2708 patients undergoing either AGB-to-LSG or AGB-to-Roux-en-Y Gastric Bypass (RYGB) conversion. Their study showcased a commendably low complication rate of 1% to 4% among those subjected to LSG conversion, attesting to the procedural viability under optimal circumstances [9].

Our presented case assumes paramount significance, as it stands as the solitary instance of a documented complication in the current body of literature. This unique complication, characterized by a biliary leak triggered by the disruption of a biliary canaliculus within the second Couinaud segment, emerged due to the adhesions between the liver and the previously placed gastric band. The manifestation of this complication occurred approximately six hours after surgery. This case report contributes significantly to our understanding of conversion surgeries following AGB placement. It underscores the importance of comprehending complications, as well as the significance of timely diagnosis and effective treatment, within this evolving field.

## Conclusions

This case report highlights the complex challenges involved in managing complications related to AGB placements. The unique biliary leak complication observed in this report, originating from the second Couinaud hepatic segment, serves as the first clinically documented case of its kind. It also underscores the necessity for careful planning, precise surgical techniques, and vigilant monitoring during the evolving landscape of revision surgeries. As the field progresses, these insights contribute significantly to optimizing patient management strategies within bariatric care.
